# Definitive chemoradiotherapy for patients with malignant stricture due to T3 or T4 squamous cell carcinoma of the oesophagus

**DOI:** 10.1038/sj.bjc.6600684

**Published:** 2003-01-28

**Authors:** K Kaneko, H Ito, K Konishi, T Kurahashi, T Ito, A Katagiri, T Yamamoto, T Kitahara, Y Mizutani, A Ohtsu, K Mitamura

**Affiliations:** ^1^Second Department of Internal Medicine, Showa University School of Medicine, 1-5-8, Hatanodai, Shinagawa-ku, Tokyo 142-8666, Japan; ^2^Department of Radiology, Showa University School of Medicine, 1-5-8, Hatanodai, Shinagawa-ku, Tokyo 142-8666, Japan; ^3^Division of Gastrointestinal Oncology/Digestive Endoscopy, National Cancer Centre Hospital East, 6-5-1, Kashiwanoha, Kashiwa, Chiba 277-8577, Japan

**Keywords:** oesophageal carcinoma, dysphagia, chemoradiotherapy, prognosis

## Abstract

We retrospectively investigated the efficacy and feasibility of concurrent chemoradiotherapy for patients with severe dysphagia caused by oesophageal squamous cell carcinoma. Concurrent chemoradiotherapy was performed in 57 patients with T3 or T4 disease containing M1 lymph node (LYM) disease. Chemotherapy consisted of protracted infusion of 5-fluorouracil (5-FU) 400 mg m^−2^ 24 h^−1^ on days 1–5 and 8–12, combined with 2-h infusion of cisplatin (CDDP) 40 mg m^−2^ on days 1 and 8. Radiation treatment at a dose of 30 Gy in 15 fractions of the mediastinum was administered concomitantly with chemotherapy. A course schedule with 3-week treatment and a 1 to 2-week break was applied twice, with a total radiation dose of 60 Gy, followed by two or more courses of 5-FU and CDDP. In all, 24 patients (42%) achieved a complete response, and the 3-year survival rate was 19%. Major toxicities were leukocytopenia and oesophagitis, and there were two (4%) treatment-related deaths. In contrast, 22 patients with T3 disease survived longer than 35 patients with T4 disease (*P*=0.001); however, the survival rate in 15 patients with M1 LYM disease did not differ significantly from that in 42 patients without M1 LYM disease (*P*=0.3545). Our results indicate that definitive chemoradiotherapy is potentially curative for locally advanced oesophageal carcinoma with malignant stricture. The efficacy and survival of patients treated with this regimen are related to the T factor.

The indications for definitive chemoradiotherapy for patients with severe dysphagia caused by oesophageal carcinoma are poorly defined. Dysphagia is a major symptom that affects the quality of life in patients with unresectable oesophageal tumours. Chemotherapy, radiotherapy, laser therapy, and stent insertion have been reported to relieve symptoms. Recently, metallic stents have become popular in the palliative treatment of patients with dysphagia caused by oesophageal carcinoma (Song *et al*, 1991; Bethge *et al*, 1992; Fleischer *et al*, 1992; Kozarek *et al*, 1992; Schaer *et al*, 1992; Knyrim *et al*, 1993; May *et al*, 1995; Wengrower *et al*, 1998). These stents can be inserted on an outpatient basis and provide rapid relief of symptoms of dysphagia. However, the indications for metallic stent insertion in patient with severe dysphagia caused by oesophageal carcinoma also remain controversial, and the clinical staging has not been evaluated before implantation of metallic stents in these reports.

On the other hand, the effects of chemotherapy combined with radiotherapy on oesophageal carcinoma have been investigated since the 1980s. Several investigators have reported successful results with these modalities, either with or without surgery, against local–regional carcinoma (Leichman *et al*, 1984; Leichman *et al*, 1987; Poplin *et al*, 1987; Coia *et al*, 1991; Frorastiere *et al*, 1993; Poplin *et al*, 1994). The combination of 5-fluorouracil (5-FU) and cisplatin (CDDP) has become a standard regimen, not only because of the clinical outcome but also because of the synergism between the two agents and their radiosensitising effects (Douple *et al*, 1980; Scanlon *et al*, 1986; Byfield, 1990). Recently published results on chemoradiotherapy indicated that it offers various advantages for the treatment of carcinoma of the oesophagus (Frorastiere *et al*, 1993; Coia, 1994). In a prospective randomised trial by the Radiation Therapy Oncology Group, which compared chemoradiotherapy with radiotherapy alone, the combined-modality arm demonstrated a significant improvement of survival (Herskovic *et al*, 1992), with a 5-year survival rate of 27%, compared with 0% for radiotherapy alone (Al-Sarraf *et al*, 1997). With regard to the indications of chemoradiotherapy as a curative treatment for patients with locally advanced diseases, our multicentre study suggested that concurrent chemoradiotherapy was potentially curative even in cases with locally advanced carcinoma of the oesophagus (i.e., T4 and/or M1 lymph node metastasis (LYM) disease) (Ohtsu *et al*, 1999). Of the 54 patients in that study, 18 (33%) achieved a complete response, and the 3-year survival rate was 23%. On the other hand, there are no studies that investigated the effects of chemoradiotherapy in patients with oesophageal stricture caused by tumours presenting with dysphagia as the principal symptom.

In the present retrospective study, we evaluated the effects of chemoradiotherapy for patients with ‘malignant stricture’ caused by squamous cell carcinoma of the oesophagus. Definition of tumour stage was based on the 1987 criteria of the tumour-node-metastasis (TNM) classification of the International Union Against Cancer (UICC). Accordingly, T4 was defined as a tumour that invaded contiguous structures and M1 LYM was defined as nodal metastasis beyond the regional lymph nodes. The efficacy and feasibility of chemoradiotherapy were analysed in consecutive patients with malignant stricture caused by oesophageal squamous cell carcinoma.

## Methods and materials

### Patient population

From May 1996 to March 2000, 70 consecutive patients aged ⩽75 years were diagnosed at Showa University School of Medicine as having locally advanced oesophageal carcinoma with malignant stricture associated with severe dysphagia. Of the 70 patients, 13 were excluded from the present study for the presence of malignant fistula (*n*=1), distant metastasis (liver metastasis (*n*=4), lung metastasis (*n*=2), bone metastasis (*n*=3)), medical conditions (including ineligible laboratory data as defined below, *n*=2), or concurrent gastric cancer (*n*=1). None of the patients had other malignancies or surgery and chemotherapy for previous diseases. Previous studies indicated that 95% of oesophageal cancers in Japanese patients are squamous cell carcinomas (Japanese Research Society for Esophageal Diseases, 1997). In agreement with that observation, none of the patients enrolled in this study had oesophageal adenocarcinoma; thus, all patients had squamous cell carcinoma of the oesophagus. All patients were enrolled in the present study after registration in the previous study (Ohtsu *et al*, 1999).

### Eligibility criteria

Patients who were eligible for this trial had previously untreated, histologically confirmed squamous cell carcinoma of the thoracic oesophagus. The tumours had to show evidence of T3 and T4 disease, containing M1 LYM disease, based on the staging criteria of the UICC. The prestudy clinical evaluation included air contrast barium oesophagography, oesophagoscopy, neck computed tomography (CT), chest CT, abdominal CT, endoscopic ultrasonography, bronchoscopy, and bone scan. However, endoscopic ultrasonography was optional since the endoscope could not be passed through stenotic lesions in most cases (89%). Bronchoscopy was performed in some cases when tracheobronchial involvement was suspected. Adjacent organs were considered to be involved if the tumours extended into the oesophageal lumen, caused deformity of the tracheobronchial tree or if the tumours appeared to be attached to the organs at a >90° angle to the thoracic aorta as observed on the CT scan (Picus *et al*, 1983; Takashima *et al*, 1991). T3 or a lesser extent of the disease was determined by endoscopic ultrasonography. In those patients who could not undergo this procedure, T3 was defined based on the lack of any associated abnormal bronchoscopic findings; that is, no deformity of the airway and tracheobronchial tree on the CT scan. Furthermore, we modified the criteria described previously for the definition of T3 disease (Picus *et al*, 1983) to include tumours attached to the organs at a ⩽90° angle to the thoracic aorta as observed on the CT scan. The patients were considered to have LYM if the tumour was ⩾1 cm in diameter (Curtin *et al*, 1998). Radiological evaluations for staging were reviewed by two radiologists (TK and YM) and the physicians at Showa University School of Medicine, as was reported in previous studies (Picus *et al*, 1983; Takashima *et al*, 1991; Curtin *et al*, 1998). However, while the UICC staging criteria were adopted in these previous studies, we used a non-standard staging technique in this study, especially when evaluating the depth of tumour infiltration. The clinical staging was also evaluated according to 1983 AJCC staging criteria. The following criteria were used for enrolment for chemoradiotherapy: (1) An Eastern Cooperative Oncology Group (ECOG) performance status (PS) of 2 or less, (2) satisfactory haematologic function (leukocyte count ⩾3000 mm^−3^ and platelet count ⩾100 000 mm^−3^), (3) satisfactory hepatic function (AST or ALT levels within three times the normal upper limit and a serum bilirubin level of less than 2.0 mg dl^−1^), (4) good renal function (creatinine level ⩽1.5 mg dl^−1^ and creatinine clearance ⩾50 ml min^−1^), (5) satisfactory pulmonary function (PaO_2_ ⩾70 mmHg), (6) normal electrocardiogram, and (7) life expectancy ⩾8 weeks. Patients with serious complications, such as a history of ischaemic heart disease, pulmonary fibrosis, or active carcinoma at another site were excluded from the study. After explaining the true disease status and predicted complications of the treatment, including the possibility of treatment-related death, each patient gave informed consent for the study. The study protocol was approved by the Human Ethics Review Committee of Showa University School of Medicine.

### Treatment schedule

Chemotherapy consisted of protracted infusion of 5-FU at a dose of 400 mg m^−^ day^−1^ on days 1–5 and 8–12, combined with a 2-h infusion of CDDP at 40 mg m^−2^ on days 1 and 8. A 10 MV radiation treatment was administered for 3 weeks (5 days/week) at 2 Gy day^−1^, concomitantly with chemotherapy. The targeted area for carcinoma of the upper and middle thirds of the oesophagus included the primary tumours with a 3-cm margin craniocaudally and any metastatic nodes with 1- to 1.5-cm margin, in the supraclavicular fossa and mediastinum. For carcinoma of the lower third of the oesophagus, the field was extended to include the perigastric nodes, while the supraclavicular fossa was excluded if the cervical nodes were found to be negative. The daily fractional dose of radiotherapy was 2 Gy administered 5 days a week. When the planned volume included both the supraclavicular fossa and upper abdominal nodes, a daily dose of 1.8 Gy was allowed. After a dose of 30 Gy, we allowed a 1- to 2-week treatment-free period. Radiotherapy was restarted on day 29 or 36, along with the same schedule of chemotherapy as described above. The treatment course included 3 weeks of radiotherapy followed by a 1- to 2-week break, and the 30 Gy course was administered twice, with a total radiation dose of 60 Gy. The irradiation techniques were initially applied in anterior and posterior opposed fields. At 40 Gy, the radiation portals were reduced to shield the spinal cord and to encompass the primary tumour craniocaudally with a 2- to 3-cm margin, usually by using an oblique opposed field. Metastatic nodes were encompassed with a 1- to 1.5-cm margin. The total radiation dose to the spinal cord was kept at a maximum of 40 Gy. The homogeneity of the dose within the planning volume was within ±10% of the prescribed dose.

Patients who were evaluated for an objective response to the above treatment received additional chemotherapy consisting of a continuous infusion of 5-FU at a dose of 800 mg m^−2^ on days 1–5 and CDDP at a dose of 80 mg m^−2^ on day 1. This treatment schedule of 1-week treatment followed by a 3- to 4-week break was only repeated once in some patients and no further treatment was applied if no disease progression was observed. When a single course consisted of treatment followed by a >5-week break, we defined the latter as interruption. All patients receiving chemoradiotherapy were monitored by neck CT, chest CT, abdominal CT, endoscopy, and air contrast oesophagography every 4–5 weeks.

### Evaluation of response and toxicity of chemoradiotherapy

For measurable lesions, the response was assessed using the World Health Organization criteria. Briefly, a complete response (CR) was defined as the complete disappearance of all measurable and assessable disease for at least 4 weeks. A partial response (PR) was defined as more than 50% reduction in the sum of the products of the longest perpendicular diameter of measurable disease for a period of at least 4 weeks. Stable disease (SD) was defined as the failure to observe CR, PR, or progressive disease for at least 4 weeks. Progressive disease (PD) was defined as a more than 25% increase in the sum of the products of the longest perpendicular diameter of measurable disease or the appearance of new lesions. To investigate the changes in the grade of dysphagia, the response of the primary tumour was evaluated using modified criteria of the Japanese Society for Esophageal Diseases (1992). For primary tumours, CR was defined as when all visible tumours, including ulceration, disappeared for at least 4 weeks, confirmed by normal endoscopic biopsy specimens. PR represented more than 50% reduction in the area of the primary tumour as observed on oesophagography. PD was considered to be an increase in the area of the tumour of more than 25%. The response was evaluated by oesophagography, oesophagoscopy, and neck, chest and abdominal CT scans during each course.

Toxicity was evaluated using the criteria defined by the National Cancer Institute Common Toxicity Criteria (NCI-CTC, version 2.0). Toxicity was assessed on a weekly basis during chemoradiotherapy and then biweekly during the subsequent chemotherapy.

The grade of dysphagia was determined by the dysphagia score as described previously (Mellow *et al*, 1985; Knyrim *et al*, 1993). A score of 0 denoted the ability to eat a normal diet, 1 denoted the ability to eat some solid food, 2 denoted the ability to eat semi-solids only, 3 denoted the ability to swallow liquids only, and 4 denoted complete dysphagia, even to saliva. Improvement of dysphagia was defined as a decrease in the dysphagia score by at least 1 point. Furthermore, we also used the PS score to evaluate any improvement/deterioration of quality of life in patients under treatment. The PS and dysphagia scores were recorded biweekly by the physician during and after these treatments.

### Statistical analysis

Follow-up evaluations after chemoradiotherapy were performed every 3 months for the first 2 years and every 6 months thereafter by endoscopy and CT scan. Differences between the two groups were calculated by the *χ*^2^ test or the Wilcoxon rank-sum test. Survival was calculated from the data at the initiation of treatment by the actuarial Kaplan–Meier method (Kaplan *et al*, 1958). Survival differences between the two groups were assessed by the log-rank test. Multivariate analyses were performed using multiple logistic regression. *P*-values of less than 0.05 were considered significant.

## Results

### Patient characteristics

The characteristics of the participating 57 patients are listed in Table 1

**Table 1:** Patient characteristics

No. of patients	57
Sex (male / female)	47/10
Age (range)	64 (45–75 years)
*Performance status*	
0	41
1	15
2	1
Median tumour length (range)	7.0 (4–15 cm)
*Location*^a^	
Upper	11
Middle	31
Lower	15
*Histopathology*	
Well differentiated	6
Moderately differentiated	41
Poorly differentiated	10
*Stage (UICC)*	
T3 M0	18
T3 M1	4
T4 M0	24
T4 M1	11

a Location of the tumour according to the TNM classification; UICC: International Union Against Cancer.

. Of these, 47 patients were men and 10 were women, and the median age was 64 years. Most patients had a good performance status. According to our criteria, the clinical staging was classified as follows: stage II in four patients, stage III in 38, and stage IV in 15. There were 22 patients (39%) with T3 disease, and 35 (61%) with T4 disease. Of 57 patients, 15 (26%) had M1 LYM disease. Clinically involved sites in the 35 cases with T4 disease were thoracic aorta (20 patients), tracheobronchial tree (13 patients), and both sites (2 patients). A single patient had cervical node metastasis, 12 had abdominal nodes, and two had metastases in both nodes. Most (90%) of the primary tumours were more than 5 cm long, with a median length of 7 cm (range, 4–15 cm). All 57 patients had histopathologically confirmed squamous cell carcinoma. In all, 53 patients (93%) completed at least the chemoradiotherapy segment with a total radiation dose of 60 Gy. The remaining four patients did not complete chemoradiotherapy; two experienced disease progression and two died because of treatment-related oesophagoaortic fistula. Of the 53 patients with complete chemoradiotherapay segment, eight and 22 patients had a 1-week break and a 2-week break after a 3-week treatment, respectively. The remaining 23 patients had an interruption during chemoradiotherapy. Of the 46 patients who responded to chemoradiotherapy, 40 (87%) received the additional two or more courses of chemotherapy. However, six patients completed only one course of chemotherapy, because these six patients achieved a CR after one course of chemotherapy.

According to the 1983 AJCC criteria, the clinical staging was classified as follows: stage II in none, stage III in 50 patients, and stage IV in seven. There were 32 patients (56%) with T3 disease, and 25 (44%) with T4 disease in AJCC criteria. None of the patients was classified as T2 disease since all patients had dysphagia. Of the 57 patients, T4 disease was predominant in our criteria; however, T3 disease was predominant in AJCC criteria. The frequency of T3 and T4 disease based on our criteria was not significantly different from that in AJCC criteria (*P*=0.0607). In contrast, 26% of 57 patients had M1 LYM disease based on our criteria; whereas 12% had M1 LYM based on AJCC criteria alone. There was no significant difference between the two groups (*P*=0.0576). Four patients were classified as stage II (T3N0M0) based on our criteria; however, none of the patients had stage II based on AJCC criteria and all patients were classified as stage III or IV.

### Clinical response to chemoradiotherapy

The results of the overall response are summarised in Table 2

**Table 2:** Response results

		**CR**		**PR**		**SD**		**PD**	
	**Number of patients**	***n***	***%***	***n***	***%***	***n***	***%***	***n***	***%***
Primary tumour	57	25	44	22	39	10	17	0	0
Overall	57	25	42	22	39	9	16	2	3

CR=complete response; PR=partial response; SD=stable disease; PD=progressive disease.

. Of the 57 eligible patients, 24 (42%) achieved CR. In all, 46 patients, including the 24 CR cases, demonstrated an objective response according to the Japanese evaluation criteria, which resulted in a response rate of 81%. Nine patients showed an SD, and two had a PD. The CR rate was significantly lower in patients with T4 (10 of 35; 29%) disease than in those with T3 (14 of 22; 64%, *P*=0.009). Furthermore, the CR rate in patients with M1 LYM (4 of 15; 27%) disease was not significantly different from that in patients with M0 LYM (20 of 42; 48%, *P*=0.2663). In 15 patients with M1 LYM disease, the CR rate in those with cervical node metastasis was not significantly different from that in patients with abdominal node metastasis. Of 11 patients with T4M1 disease, two (18%) achieved a CR and one (9%) had over 3-year survival. In multivariate analyses, the CR rate based on our criteria was related to T factor (*P*=0.0351), but not to M factor (*P*=0.4413). In contrast, the CR rate based on AJCC criteria did not correlate with the T (*P*=0.0535) or M factor (*P*=0.6805).

### Toxicity

The major side effects of chemoradiotherapy encountered in our patients during treatment are listed in Table 3

**Table 3:** Major complications appearing during and after chemoradio- therapy

	**Grade 1**		**Grade 2**		**Grade 3**		**Grade 4**	
	***n***	***%***	***n***	***%***	***n***	***%***	***n***	***%***
Leukocytopenia	11	19	23	40	14	25	3	5
Anaemia	3	5	22	39	19	33	0	0
Thrombocytopenia	5	9	6	11	5	9	3	5
Nausea/Vomiting	17	30	5	9	9	16	0	0
Diarrhoea	5	9	11	19	5	9	2	4
Mucositis	6	11	6	11	5	9	0	0
Oesophagitis	23	40	11	19	9	16	5	9
Renal	5	9	11	19	0	0	0	0
Pulmonary^a^	11	19	5	9	0	0	0	0
Cardiac^a^	0	0	6	11	1	2	0	0

a Late radiation-related complications.

. These included myelosuppression and oesophagitis. Grade 3 and higher leukocytopenia, anaemia, thrombocytopenia, and oesophagitis occurred in 30, 33, 14, and 25% of the patients, respectively. Two patients (4%) developed sepsis associated with leukocytopenia; however, these patients recovered from sepsis with the use of filgrastim and antibiotics. Of 35 patients with T4 disease, three (9%) developed treatment-related perforation of the oesophageal wall: one developed a mediastinal fistula, while each of the other two developed an aortic fistula. These three patients had T4 disease before treatment, and these events occurred during chemoradiation. One patient showed spontaneous healing of the mediastinal fistula after the disappearance of inflammatory findings, despite continuation of treatment, and achieved a PR. The remaining two patients with oesophagoaortic fistulae died of massive bleeding during two courses and four courses of chemoradiation, respectively. Nausea was not associated with chemoradiotherapy for patients with abdominal metastatic nodes.

Treatment was interrupted during the chemoradiotherapy segment in 23 patients for the following reasons: persistent leukocytopenia (*n*=17), fistula (*n*=3), and other complications (*n*=3). The median duration of the interruption because of persistent leukocytopenia was 6 days (1–13 days) after a definite break. All 17 patients could pursue the treatment. There were two (4%) deaths related to treatment: as a result of oesophagoaortic fistula, as mentioned above. However, the patient with mediastinal fistula continued the treatment after a 1-week interruption, since inflammation could be improved with the use of antibiotics. In all patients, treatment had little effect on body weight (−1.3±9.2%).

Late radiation-related complications included pneumonitis and pericarditis. Pneumonitis of grade 2 or less occurred in 16 patients (28%), but none of the patients developed grade 3 or higher toxicity. Pneumonitis occurred at a median of 5.5 months from the end of radiotherapy (range, 3–8 months). Grade 3 pericarditis with pericardial effusion was detected in one patient (2%). Dyspnoea owing to pericardial effusion appeared after approximately 6 months from the end of radiotherapy. Histopathologically, no malignant cells were found in pericardial effusion samples. This patient remains disease-free and is still alive after more than 3 years of termination of treatment.

### Grade of dysphagia

Table 4

**Table 4:** Dysphagia score before and after concurrent chemoradiotherapy

	**Before treatment**		**After treatment**	
**Dysphagia score**	***n***	***%***	***n***	***%***
Grade 0	0	0	24	42
Grade 1	0	0	16	28
Grade 2	6	10	8	14
Grade 3	36	65	7	12
Grade 4	15	25	2	4

A score of 0 denotes the ability to eat a normal diet, 1 denotes the ability to eat some solid food, 2 denotes the ability to eat semisolids only, 3 denotes the ability to swallow liquids only, and 4 denotes complete dysphagia, even to saliva.

summarises the effects of treatment on the dysphagia score. Most patients had severe dysphagia caused by oesophageal carcinoma. With regard to the primary lesions as evaluated by the Japanese criteria, 47 (83%) of the 57 patients showed improvement of the dysphagia score, including 25 (44%) with CR. These 25 CR cases became dysphagia-free, and 11 (44%) never complained of dysphagia over a 3-year period. Of the remaining 13 patients, dysphagia appeared again after treatment in eight, which was because of local recurrence in five, and compression of metastatic lymph node in three. Implantation of a self-expanding metallic stent was performed in these patients, because these eight patients developed dysphagia caused by malignant stricture after failure of chemoradiotherapy.

The dysphagia score decreased from 3.2 to 1.1 (*P<*0.0001), and dysphagia improved in 46 (81%) of the 57 patients. Of the 47 patients in whom the primary lesion responded to chemoradiotherapy, dysphagia improved in 46, while one patient could not take solid food because of a progressive oesophageal stricture induced by radiation-related fibrotic changes. Of these 46 patients, dysphagia improved in 27 (59%) following a single course of chemoradiotherapy, and in 19 (41%) after two courses. The median duration of dysphagia improvement was 10 months after treatment in these patients. The proportion of PS 0, 1, 2, and 3 before treatment was 41 (72%), 15 (26%), 1 (2%), and 0 (0%), respectively. The proportion of PS 0, 1, 2, and 3 after treatment was 37 (65%), 16 (28%), 3 (5%), and 1 (2%), respectively. The PS was still 2 or 3 after treatment in some patients with progressive disease; however, most patients had a PS before and after treatment.

### Survival

**Figure 1:**
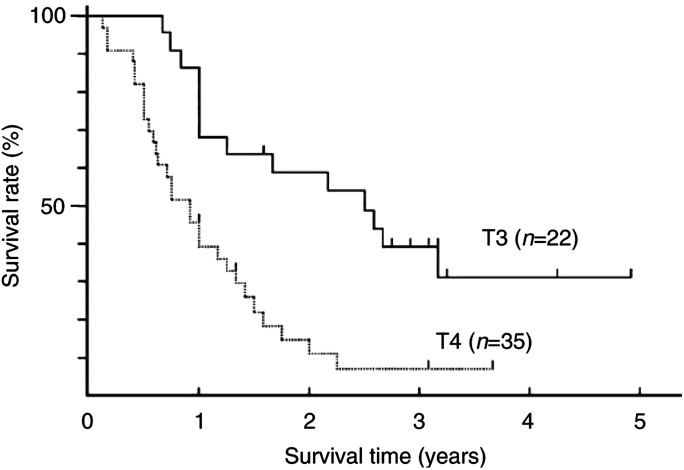
Survival curves of 22 patients with T3 disease and 35 patients with T4 disease.

**Figure 2:**
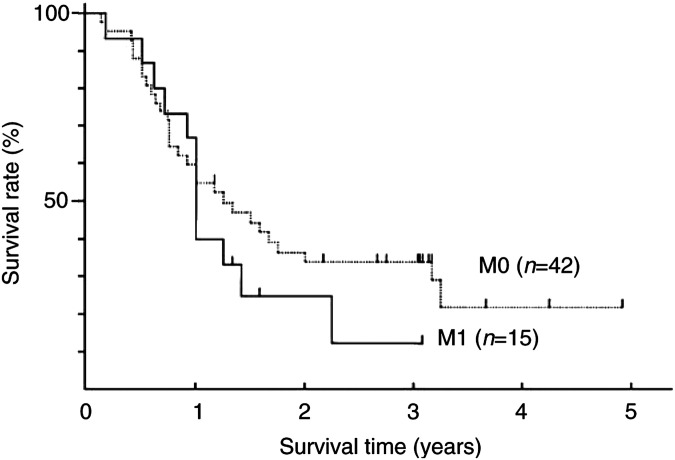
Survival curves of 42 patients with M0 disease and 15 patients with M1 LYM disease.

After a median follow-up period of 14 months (range, 1–58 months), 17 (30%) patients were still alive. Survival rates of 1 and 3 years were 61% (35 of 57) and 19% (11 of 57), respectively. Figure 1

shows the survival curves of 57 patients based on the T factor (T3 or T4). The median survival times of 22 patients with T3 disease and 35 patients with T4 disease were 29 and 11 months, respectively; the survival rate of patients with T3 disease was significantly longer than that of patients with T4 disease (*P*=0.001, log-rank test). Furthermore, when the patients were divided into two subgroups of 15 with M1 LYM (four patients with T3 and 11 with T4) and 42 with M0 LYM (18 patients with T3 and 24 with T4), the median survival time of 42 patients with M0 disease (15 months) was not different from that of 15 patients with M1 disease (12 months, *P*=0.3545, log-rank test). Multivariate analyses showed that the survival rate based on our criteria was strongly related to T factor (*P*=0.0051), but not to M factor (*P*=0.4615). In contrast, the proportion of T3 and T4 disease or M0 and M1 disease classified based on our criteria was not similar to that classified by AJCC criteria, although the survival rate based on the AJCC criteria was also related to the T factor (*P*=0.0247) but not to the M factor (*P*=0.5128) in multiple logistic regression (see Figure 2

).

## Discussion

The value of chemoradiotherapy for the treatment of unresectable oesophageal carcinoma remains controversial, and only a few clinical studies have been published since the 1980s (Leichman *et al*, 1984; Leichman *et al*, 1987; Poplin *et al*, 1987; Coia *et al*, 1991; Herskovic *et al*, 1992; Frorastiere *et al*, 1993; Poplin *et al*, 1994). Most patients in these studies had local–regional disease (UICC stage I or II). Although some reports included patients with T4 disease, the proportion of such patients was usually low and the clinical outcomes were not clearly described. The results of these studies, including CR rates and survival rates, were confusing, since the clinical and pathological backgrounds varied, especially with regard to the stage of the disease. Therefore, stratification by clinical stage should be applied when evaluating the impact of treatment on survival and response. Coia *et al* (1991) reported long-term results with 5-FU and mitomycin C chemotherapy combined with radiation therapy. In their study, 33 patients with stage III and IV disease were treated with chemotherapy and 50 Gy of radiation therapy with palliative intent; this treatment resulted in a median survival duration of 9 months and a 2-year survival rate of only 3% (Coia, 1994). Zeone *et al* (1992) reported the results of curative nonsurgical treatment that consisted of 5-FU, CDDP, and 64 Gy of radiotherapy combined with neodymium: yttrium-aluminium garnet (Nd : YAG) laser therapy in appropriate patients. They treated 65 patients who had predominantly T1–3 disease, but their study included five patients with T4 disease. Although the 3-year survival rate of the 65 eligible patients was 37%, all five patients with T4 disease died within 18 months. In another large study from Australia, 79 patients with advanced-stage carcinoma, including 25 with systemic metastasis, were treated with 5-FU, CDDP, and 30–35 Gy of radiation therapy (Burmeister *et al*, 1995). A 3-year survival rate of 9% was achieved in patients with advanced disease. However, clinical stages based on the TNM classification were not described; therefore, the stage at which patients survived longer is unknown. A literature search produced no other studies that specifically investigated chemoradiotherapy for locally advanced disease, such as T4 and/or M1 LYM. In our previous study, a CR rate of 33% and a 3-year survival rate of 23% were achieved in patients with unresectable T4 tumours and/or M1 LYM disease (Ohtsu *et al*, 1999), suggesting that concurrent chemoradiotherapy was potentially curative for locally advanced carcinoma. In our present study, a CR rate of 39% and a 3-year survival rate of 19% were achieved in patients with ‘severe dysphagia’ accompanied by T3 or T4 disease.

With regard to the efficacy and feasibility of chemoradiotherapy, our clinical outcomes in the present study were similar to those in our previous multicentre study. In our previous and present studies, the extended field of irradiation was used to cover the three field dissected areas by extended surgery in Japan. Furthermore, a combination of 5-FU and CDDP has become a standard regimen because of the synergism between the two agents and their radiosensitising effects (Douple *et al*, 1980; Scanlon *et al*, 1986; Byfield, 1990).

Some studies of a continuous irradiation course combined with 5-FU and CDDP indicated that grade 3 and higher leukocytopenia and oesophagitis occurred in 33–54% and 48–50% of patients, respectively (Herskovic *et al*, 1992; Poplin *et al*, 1994). It is likely that severe leukocytopenia and oesophagitis frequently occurred in a continuous irradiation course. Since the presence of severe toxicity because of both the extended field of irradiation and a combination of chemoradiotherapy had been expected, we used a split course radiation technique with a 1- to 2-week treatment-free period instead of using a continuous irradiation course. In contrast, the periods of recovery from toxicity were not sufficient for a 1-week break in many patients. Therefore, we believed that at least a 2-week break would be required to administer chemoradiotherapy without an interruption. Our results suggested that definitive chemoradiotherapy with a split course radiation technique accompanied by a 2-week break is feasible for locally advanced carcinoma.

In contrast, our results were associated with significant toxicity, consisting predominantly of leukocytopenia and perforation of the oesophageal wall. The high incidence of leukocytopenia and oesophagitis might be because of both the extended field of irradiation and combination of chemoradiotherapy. Fortunately, the leukocytopenia was not a fatal complication, and patients with leukocytopenia-related sepsis could recover with the use of filgrastim and antibiotics. Perforation of the oesophageal wall was an unavoidable significant toxic effect of treatment for T4 disease. However, no perforation occurred in patients with T3 disease. Previous studies reported the development of fistula in 29% of 94 patients with oesophagobronchial involvement who were treated with radiation therapy alone (Roussel *et al*, 1995). Early death occurred in all patients who developed complications, with a median period of 1.7 months. The rate of oesophageal perforation in the present study was 9% (three of 35 patients) in patients with T4 disease, which was lower than that reported by Roussel *et al*. Furthermore, one of the three perforations in our study closed spontaneously following additional chemoradiotherapy after improvement of the inflammatory process, and the patient achieved PR with respect to the primary tumour. Insertion of the metallic stent as a palliative treatment was not performed in our patients despite perforation of the oesophageal wall. However, these results do not support the criticism that chemoradiotherapy should be contraindicated for T4 disease, especially in cases that involve fistula.

In contrast, several reports have proposed that insertion of a metallic stent is effective in the palliative treatment of malignant oesophageal stricture (Song *et al*, 1991; Bethge *et al*, 1992; Fleischer *et al*, 1992; Kozarek *et al*, 1992; Schaer *et al*, 1992; Knyrim *et al*, 1993; May *et al*, 1995; Wengrower *et al*, 1998). However, the indication for metallic stent insertion in patients with malignant stricture caused by oesophageal carcinoma remains controversial. In one study, 21 patients with malignant oesophageal stricture received a self-expanding metallic stent, and all stents were placed successfully with no immediate severe complications (Knyrim *et al*, 1993). Dysphagia improved in 92% of their patients and the dysphagia score decreased from 3 to 1, while the mean survival time was 168 days. In another study, 30 patients with incurable malignant obstruction of the oesophagus and cardia were treated with self-expanding metallic stents (May *et al*, 1995). All stents were placed successfully with no early complications. Dysphagia improved in 83% of the patients within 1 week, and the mean survival time was 108 days (range, 14–211 days). In a multicentre study from Israel, 81 patients with malignant obstruction of the oesophagus and gastric cardia were treated with self-expanding metallic coils (Wengrower *et al*, 1998). All coils were placed successfully and dysphagia improved in 96% of the patients, while the dysphagia score dropped from 3.5 to 1.2. The mean survival time was 16 weeks (range, 4–56 weeks). In these results (Knyrim *et al*, 1993; May *et al*, 1995; Wengrower *et al*, 1998), the stent was placed successfully and dysphagia improved in approximately 90% of the patients. Furthermore, there seemed to be few fatal complications in the early postinsertion period, although late complications occurred in 22–30% of the patients (Knyrim *et al*, 1993; May *et al*, 1995; Wengrower *et al*, 1998). The mean survival time was 4–5 months after stent insertion. Efficacy, complications, and survival time are not likely to be different, although different stents are inserted as an initially palliative treatment. Approximately 90% of our patients had severe dysphagia before chemoradiotherapy, however, dysphagia improved in 81% of the patients and the average dysphagia score decreased from 3.1 to 1.1. Most of our patients had a good PS before and after treatment. The change in the grade of dysphagia in our study was not different from those reported by other investigators (Knyrim *et al*, 1993; May *et al*, 1995; Wengrower *et al*, 1998). However, implantation of the metallic stent is unlikely to be curative in patients with severe dysphagia. In contrast, most patients with T3 disease become dysphagia-free following such a procedure. If a metallic stent is initially inserted for patients with severe dysphagia, such patients, especially those with T3 disease, are unlikely to have a complete cure. Assessment of the clinical stage according to the TNM classification should be performed in patients with dysphagia caused by advanced oesophageal carcinoma, and chemoradiotherapy should be provided to patients with T3 or T4 accompanied by M1 LYM disease. Insertion of a metallic stent as a palliative treatment may be of limited value, for example, to patients with oesophageal fistula or systemic metastasis, or those who develop stricture after failure of the primary curative treatment (Kaneko *et al*, 2002).

The development of imaging techniques such as CT scan, magnetic resonance imaging, and endoscopic ultrasonography, has allowed a more accurate clinical staging of oesophageal tumours in recent years. These advances in diagnostic procedures have allowed the selection of optimal treatment modality through clinical staging. Although there are some ‘grey zones’ with respect to determining T3 or T4 disease by imaging, several studies have reported the successful use of CT scans and/or magnetic resonance imaging with an accuracy rate of ⩾80% (Picus *et al*, 1983; Takashima *et al*, 1991). We adopted their reported criterion to define T3 or T4 disease. Diagnostic radiologists, together with medical oncologists, were responsible for the final staging. With regard to the determination of positive nodes, there are no reliable diagnostic staging criteria to date. Therefore, we used the 1-cm size to indicate a positive node. The positive predictive value of this criterion was only 50% in the study of Curtin *et al* (1998). This low value might explain our good results in cases with M1 LYM disease, although all 15 patients with M1 LYM disease had advanced T3 or T4 disease with severe stricture. Moreover, the patients classified with T3M0 and T4M0 disease would have had occult nodal involvement that was not detected by CT scan. Since the survival rate in patients with M0 disease did not differ significantly from that of patients with M1 LYM disease (*P*=0.3545), it is likely that any attempt to separate these groups would be rather artificial. In contrast, only 11% of patients had an EUS to define the depth of wall penetration to establish T3 disease. Since our staging criteria for T3 disease used before treatment is not standard, the clinical staging was also performed according to 1983 AJCC staging criteria. The proportions of T3 and T4 based on the AJCC criteria were not similar to that based on our criteria, although no significant differences were noted between the two groups. However, our results suggested that the survival rate was significantly related to T factor in multivariate analyses, when the clinical staging is classified according to either our criteria or AJCC criteria. The prior staging systems (1983 AJCC) were based on clinical information, and the system may remain an important prognostic indicator for patients managed with chemoradiation.

Literature search did not find other reports that recommended chemoradiotherapy for locally advanced carcinoma with severe stricture. In the present retrospective study, we investigated the efficacy and feasibility of concurrent chemoradiotherapy for patients with severe dysphagia caused by advanced oesophageal squamous cell carcinomas of T3 or T4 and M1 LYM disease. Our results, especially with regard to long-term survival, allow us to suggest that definitive chemoradiotherapy is potentially curative for locally advanced oesophageal carcinoma defined by clinical imaging. Thus, proper clinical staging before treatment is important in patients with dysphagia caused by oesophageal carcinoma, since the efficacy of such treatment correlates with the T factor. Treatment-related fatal complications can occur in patients with T4 disease; however, toxicities including late radiation-related complications were manageable. Further investigation of the combined treatment modality as a curative approach is required particularly for cases with locally advanced oesophageal carcinoma with T4 disease.
